# Electrocardiogram characteristics prior to in-hospital cardiac arrest

**DOI:** 10.1007/s10877-014-9616-0

**Published:** 2014-09-19

**Authors:** Mina Attin, Gregory Feld, Hector Lemus, Kayvan Najarian, Sharad Shandilya, Lu Wang, Pouya Sabouriazad, Chii-Dean Lin

**Affiliations:** 1 School of Nursing, San Diego State University, 5500 Campanile Drive, San Diego, CA 92182 USA; 2 Department of Medicine, Cardiology Division, Electrophysiology Section, University of California, San Diego, San Diego, CA USA; 3 School of Public Health, San Diego State University, San Diego, CA USA; 4 Department of Computational Medicine and Bioinformatics, Michigan Center for Integrative Research in Critical Care, University of Michigan, Ann Arbor, MI USA; 5 Department of Emergency Medicine, Michigan Center for Integrative Research in Critical Care, University of Michigan, Ann Arbor, MI USA; 6 SciCore Technology, Richmond, VA USA; 7 Department of Bioengineering, College of Engineering, San Diego State University, San Diego, CA USA; 8 Department of Mathematics and Statistics, San Diego State University, San Diego, CA USA; 9 Department of Computer Science and Engineering, Michigan Center for Integrative Research in Critical Care, University of Michigan, Ann Arbor, MI USA

**Keywords:** In-hospital cardiac arrest, Asystole, Pulseless electrical activity, Electrocardiogram

## Abstract

Survival after in-hospital cardiac arrest (I-HCA) remains < 30 %. There is very limited literature exploring the electrocardiogram changes prior to I-HCA. The purpose of the study was to determine demographics and electrocardiographic predictors prior to I-HCA. A retrospective study was conducted among 39 cardiovascular subjects who had cardiopulmonary resuscitation from I-HCA with initial rhythms of pulseless electrical activity (PEA) and asystole. Demographics including medical history, ejection fraction, laboratory values, and medications were examined. Electrocardiogram (ECG) parameters from telemetry were studied to identify changes in heart rate, QRS duration and morphology, and time of occurrence and location of ST segment changes prior to I-HCA. Increased age was significantly associated with failure to survive to discharge (*p* < 0.05). Significant change was observed in heart rate including a downtrend of heart rate within 15 min prior to I-HCA (*p* < 0.05). There was a significant difference in heart rate and QRS duration during the last hour prior to I-HCA compared to the previous hours (*p* < 0.05). Inferior ECG leads showed the most significant changes in QRS morphology and ST segments prior to I-HCA (*p* < 0.05). Subjects with an initial rhythm of asystole demonstrated significantly greater ECG changes including QRS morphology and ST segment changes compared to the subjects with initial rhythms of PEA (*p* < 0.05). Diagnostic ECG trends can be identified prior to I-HCA due to PEA and asystole and can be further utilized for training a predictive machine learning model for I-HCA.

## Introduction

In-hospital cardiac arrest (I-HCA) accounts for approximately 200,000 cardiac arrests in the United States each year [1] and survival to discharge after cardiopulmonary resuscitation is less than 30 % [2]. Overall predictors of poor survival among hospitalized subjects with I-HCA include age, metastatic malignancy, impaired renal function and dependent functional status [3]. However it is not clear whether I-HCA patients with known heart diseases have similar survival predictors.

Approximately 70 % of initial rhythms in patients with I-HCA are pulseless electrical activity (PEA) and asystole, and these rhythms are associated with higher mortality rates as compared to initial rhythms of ventricular tachycardia and fibrillation [4–6]. Telemetry use in-hospital is recognized as an independent predictor of survival to discharge regardless of rhythm prior to I-HCA [7]. Although electrocardiogram (ECG) predictors of ventricular tachycardia and ventricular fibrillation are well-studied, ECG predictors of PEA and asystole are not. Identifying ECG changes prior to PEA and asystole in patients with known heart diseases may provide diagnostic clues that will lead to prevention or more timely treatments of I-HCA in such cases. For example, the chance of survival to hospital discharge is reportedly doubled among patients who receive cardiopulmonary resuscitation (CPR) within the first minute after cardiac arrest in comparison with those who received CPR after more than a minute had elapsed [8]. Thus, our study was designed to determine whether ECG indicator(s) may predict I-HCA due to PEA or asystole in patients with cardiovascular diseases, which then might be used to shorten response time and improve treatment to increase the likelihood of successful resuscitation and discharge from the hospital.

## Methods

### Study design and setting

A retrospective study was conducted among subjects who experienced I-HCA with an initial rhythm of PEA or asystole in the medical and surgical intensive care units (ICU) and step-down units of three acute care hospitals. All I-HCA subjects were on continuous ECG monitoring (e.g., five lead ECG telemetry) prior to I-HCA, per standard of care required by each hospital, for the entire duration of stay of these patients in the ICU or step down units.

### Study population

Subjects who experienced I-HCA and underwent cardiopulmonary resuscitation (CPR) were screened from February 2010 to June 2012 for eligibility in the study. Subjects were included if they were ≥21 years of age and had a history of structural heart diseases (see definitions below). If a subject had more than one I-HCA, only the first episode of I-HCA was included in the analysis. Subjects were excluded from the study if resuscitation was initiated out-of-hospital, in the emergency room, in a procedure or operating room, or for trauma, drug overdose, hypothermia, drowning, or if they had an active cancer (e.g., receiving chemo or radiation therapy) or were comatose. Clinical characteristics of subjects were obtained from the medical record. Laboratory data including potassium, calcium, hemoglobin, blood urea nitrogen and creatinine were obtained within 24–48 h of I-HCA, and troponin and B-type natriuretic peptide (BNP) levels were recorded if obtained during the same hospitalization in which I-HCA occurred. A list of cardiac medications, sedatives, and narcotics administrated within 72 h of I-HCA was obtained. Cardiac risk factors including a history of diabetes, hypertension, and hyperlipidemia were noted. The ejection fraction (EF) from any echocardiogram obtained within 6 months prior to I-HCA was recorded. All available 12-lead ECGs prior to I-HCA were examined for evidence of bundle branch blocks.

### ECG measurements

The full disclosure ECG prior to I-HCA from continuous monitoring systems was obtained. The full disclosure ECG was recorded at a paper speed of 25 mm/s, at 10 mm/mV gain, and typically consisted of 7–8 ECG leads (i.e. I, II, III, aVR, aVL, aVF, V_1_, V_6_). Since lead V_6_ was not routinely recorded in the majority of subjects, only seven leads were used for analysis. Each full disclosure ECG was 10 s long. Full disclosure ECGs were analyzed for 2 min at the time of I-HCA and for any significant rhythm changes within 1 h of documented I-HCA. Additionally, full disclosure ECG papers were analyzed for 2 min in each of the 8 h prior to I-HCA. All rhythms prior to I-HCA were analyzed except for paced rhythms.

Heart rate was reported as the R–R interval. A decreased heart rate was defined as a decline in heart rate ≥15 beats/min at any time within 15 min of cardiac arrest. QRS duration was measured from the beginning of the Q wave (or R wave in the absence of Q wave) to the end of the S wave in each lead of the full disclosure ECG and the mean QRS duration for each lead was calculated over three beats in each full disclosure ECG paper. QRS prolongation was defined as an increase in QRS duration ≥20 ms (ms) observed within 7 h prior to I-HCA. QRS duration prior to I-HCA was noted in two ways: (1) a single longest QRS duration in any available lead for each hour and (2) the longest mean QRS duration in any available lead for each hour.

ST segment elevation or depression of ≥1 mm and QRS morphology including notching, fractionated QRS (fQRS), rSR′ and rSr′ in any available ECG lead were identified visually by experts including a physician (cardiac electrophysiologist) and the primary author. We reported only those changes in QRS morphology and ST segment elevation and depression that were not present initially at 8 h prior to arrest. The time between I-HCA and changes in QRS morphology, ST segment elevation and depression were obtained by recording the earliest change that was present in any ECG lead. The ECG locations of the earliest changes and subsequent changes were also recorded. Subjects with left bundle branch block (LBBB) confirmed through 12 lead ECG were not included in the sample set for detecting ST segment changes.

Research assistants were trained to review and evaluate ECG changes, and the correlation of measurements between research assistants and primary investigators was >90 %. A cardiac electrophysiologist was available as a consultant to review the ECG data. Additionally, we digitized ECG paper recordings for 39 subjects and performed automated measurements including heart rate [9, 10]. The agreement between manual and automated measurements was 93 %.

### Definitions

For this study, cardiac arrest was defined as a sudden cessation of cardiac function, precipitated by pulseless electrical activity or asystole (ventricular tachycardia and ventricular fibrillation were excluded) [7]. The presence of underlying cardiovascular disease was defined as documented coronary artery disease, history of percutaneous coronary intervention or coronary artery bypass graft surgery, pathological Q waves on 12 lead ECG, diagnosis of prior MI, history of valvular heart disease, aortic aneurysm, or cardiomyopathy. The presence of congestive heart failure (CHF) was confirmed by physician documentation of signs and symptoms of heart failure during the period of hospitalization in which the I-HCA occurred [11], and by echocardiogram showing left ventricular systolic dysfunction (EF < 50 %) or diastolic dysfunction [12]. In order to be included in the study, all subjects had to have at least one documented structural heart disease as described.

### Statistical analyses

Descriptive statistics were reported as means, standard deviation, and standard errors for continuous variables. Categorical variables were expressed in numbers and percentages. The Chi-squared test was used to determine the statistical significance of differences among categorical/continuous variables (e.g., ECG morphological changes among cases).

Logistic regression was used to assess association between immediate outcomes of cardiac arrest, survival to discharge, and ECG changes (e.g. changes in heart rate and QRS duration) and demographic variables. The change in heart rate within 15 min prior to I-HCA for all subjects was calculated. ANOVA and Kruskal–Wallis tests were used to detect significant differences in heart rate and QRS duration within 8 h of cardiac arrest and their comparisons with the last hour of cardiac arrest for all subjects. Statistical analyses were performed using SAS software (Version 9.3; SAS Institute Inc, Cary, NC), and Python. A *p* value <0.05 was considered statistically significant.

## Results

### Demographics

During the study, 39 of 80 subjects with I-HCA from intensive care and step-down units met inclusion criteria. Clinical characteristics are summarized in Table 1. Although 25 subjects (64 %) were resuscitated from I-HCA, 17 died in hospital before discharge, and eight survived to hospital discharge. The mean length of stay for subjects who died in hospital was 9 ± 6 days and for subjects who were discharged home 18 ± 8 days.

**Table 1:** Characteristics of subjects who experienced I-HCA due to PEA and asystole

Characteristics	All subjects (N = 39)
Age [mean (SD), year]	69.5 (13)
Male sex [no. (%)]	25 (64)
Location of cardiac arrest—ICU [no. (%)]	17 (44)
Initial rhythm of cardiac arrest—asystole [no. (%)]	15 (38)
Ejection fraction (n = 31), mean (SD)^a^	51.9 (16.2)
Body mass index (n = 36), mean (SD)^a^	28 (8)
*Risk factors [no. (%)]*	
Hypertension	33 (87)
Diabetes	16 (42)
Hyperlipidemia	19 (50)
*Medical history [no. (%)]*	
Coronary artery disease	29 (76)
Congestive heart failure	15 (39)
Coronary artery bypass graft	7 (18)
Cardiomyopathy	3 (8)
*Laboratory values, mean (SD)*	
Potassium (mEq/L)	4.4 (0.9)
Calcium (mg/dL)	8.4 (0.8)
Hemoglobin (g/dL)	10.6 (2.1)
Blood urea nitrogen (mg/dl)	40.6 (23.4)
Creatinine (mg/dl)	2.7 (2.1)
*Medications [no. (%)]*	
Beta blockers	14 (37)
Calcium channel blockers	10 (26)
ACE inhibitors	9 (23)
Vasopressors	13 (34)
Digoxin	1 (2)
Diuretics	13 (34)
Opioid	18 (47)
Benzodiazepine	11 (29)

^a^Not all subjects had complete data and the available information of subjects in each category was described by (n=). Data were expressed in numbers, percentage, mean and standard deviation

Troponin I (μg/mL) values were available in 26 subjects (67 %), of whom 16 (62 %) had elevated levels (ranging from 0.23 to 46.77). Brain natriuretic peptide levels were available in 18 subjects (46 %) with a median of 801 pg/mL (absolute median deviation = 418.5).

### Survival from cardiac arrest

Among covariates analyzed for all subjects, only older age and high creatinine were negatively associated with resuscitation from I-HCA (*p* < 0.05) and only older age was significantly associated with poor survival to discharge (*p* < 0.05). Covariate analysis found no significant association between demographic values, heart rate changes or QRS prolongation.

### Changes in heart rate and QRS prolongation prior to cardiac arrest

**Fig. 1:**
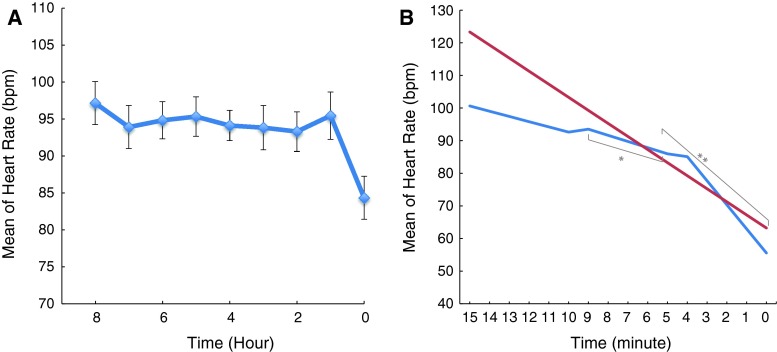
Decreased heart rate prior to cardiac arrest among all subjects: **A** Mean of heart rate within 8 h of cardiac arrest. There was a significance difference between the last hour of cardiac arrest as compared with the previous 8 h. **B** *Red color* shows the mean of heart rate within 15 min; *blue color*-within 15–10 min, 10–5 min (*p < 0.01), and 5–0 min, (**p < 0.01). Time “0” is the time of cardiac arrest

There were 32 subjects (82 %) who had decreased heart rate prior to cardiac arrest. Figure 1A, B demonstrates the mean heart rate during each of the 8 h prior to I-HCA and within 15 min of cardiac arrest in all subjects. There was a significant difference in heart rate during the last hour prior to I-HCA compared to the previous hours (*p* < 0.05) as supported by parametric (ANOVA) and non-parametric (Kruskal–Wallis) multiple comparison tests. Pairwise hourly comparisons showed that the hour prior to I-HCA contributed to the significance of the tests. Mean heart rate decreased 3.0 beats per min (bpm) within the last hour prior to I-HCA (*p* < 0.01), 4.6 ± 2.5 bpm within 15–10 min (*p* = 0.24), 5 ± 0.5 bpm within 10–5 min (*p* < 0.01) and 7.6 ± 1.3 bpm within 5–0 min (*p* < 0.01). There was no difference with respect to the change in heart rate within the last hour or 15 min prior to I-HCA in patients with an initial rhythms of PEA or asystole. Different cardiac arrhythmias during the period of decreased heart rate were observed including sinus bradycardia, with and without first degree atrioventricular (AV) block (47 %), junctional rhythm (28 %), atrial fibrillation (19 %), and third degree AV block (6 %).

Nine subjects (28 %) had a sudden decrease in heart rate over 10–25 s during the 15 min prior to I-HCA. This sudden decrease in heart rate was either followed by a continuous downtrend of heart rate or an increase in heart rate to the previous rate followed by a downtrend of heart rate leading up to I-HCA. The maximum and minimum heart rates were 100 and 38 bpm respectively, and the range of difference in heart rate was 21–60 bpm with a median of 34 in subjects with asystole and PEA. Junctional rhythms, progression of first degree to third degree atrioventricular block, and sinus bradycardia with premature atrial contractions were observed during periods with a sudden decrease in heart rate.

QRS prolongation was observed in 20 (51 %) subjects, including 19 in the group with decreased heart rate prior to I-HCA. QRS prolongation (*p* < 0.01) was observed within the last hour prior to I-HCA compared to previous hours among all subjects. QRS prolongation was significant for both the longest mean QRS and the single longest QRS measured in full disclosure ECG leads. There was no significant difference between the longest mean QRS and the single longest QRS in any ECG lead.

### QRS morphology and ST segment changes prior to cardiac arrest

**Fig. 2:**
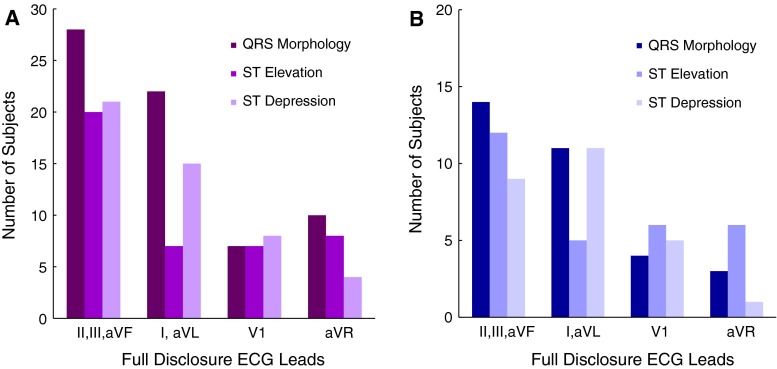
QRS morphology or ST segment changes among all subjects: **A** QRS morphology or ST segment changes in subjects with PEA or asystole. **B** Number of subjects in the ECG lead territory first to show changes in QRS morphology or ST segment deviation prior to PEA or asystolic cardiac arrest

**Fig. 3:**
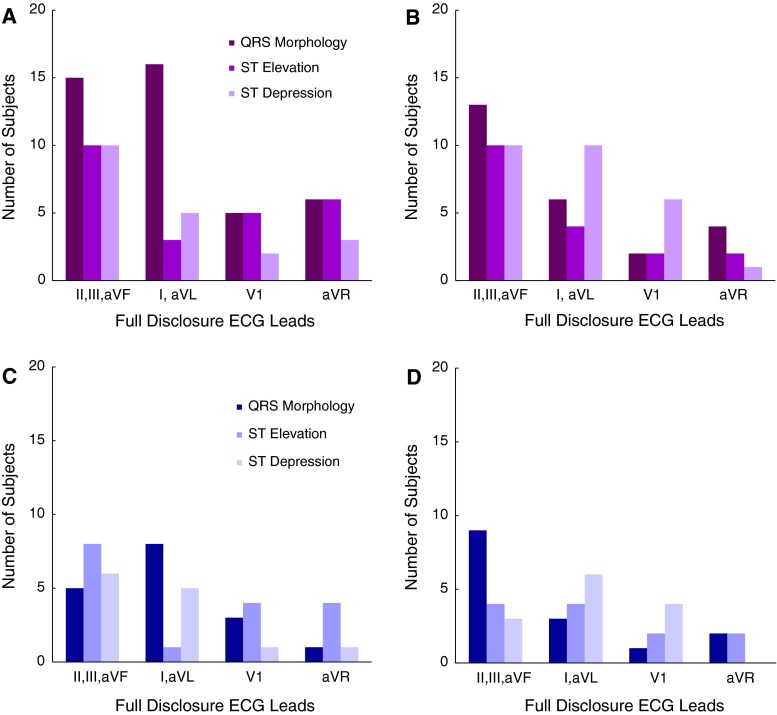
QRS morphology or ST segment changes in asytole or PEA group: **A** Asystole group, **B** PEA group, **C** Number of subjects in the ECG lead territory, first to show changes prior to asystolic cardiac arrest, **D** Number of subjects in the ECG lead territory first to show changes prior to PEA

The distribution of QRS morphology and ST segment changes in all subjects is shown in Table 2. The Fig. 2 demonstrates changes with respect to the ECG leads among all subjects with PEA and asystole. Furthermore, subjects were separated including those with an initial rhythm of PEA, and those with initial rhythm of asystole. The results of ECG Changes in subjects with initial rhythms of PEA and asystole are demonstrated in Fig. 3. Table 2 demonstrates the significant differences between these subjects with initial rhythms of PEA and asystole.

**Table 2:** Distribution of QRS morphology and ST segment changes prior to cardiac arrest

	Initial rhythms: asystole (n = 15)	Initial rhythms: PEA (n = 24)	Initial rhythms of asystole and PEA	*p* value*
QRS morphology (%)	12 (80)	9 (38)	21 (54)	0.01
ST elevation (%)	11 (73)	9 (38)	20 (51)	0.02
ST depression (%)	10 (67)	13 (54)	23 (59)	0.44
ST elevation or depression (%)	10 (67)	8 (33)	18 (46)	0.04

* Chi-square was performed to detect significant differences between PEA and asystole groups (*p* < 0.05)

The median time to I-HCA for the earliest changes in QRS morphology, ST depression and ST elevation was 121 min, 13.5 min and 6.5 min, respectively. ST segment elevation (1–5 mm) and ST depression (1–4 mm) were observed in seven subjects (18 %). The median time for the earliest transient changes in QRS morphology, ST depression and ST elevation was 14, 4, and 5 min prior to I-HCA, respectively.

If there was more than one change in QRS morphology (e.g. notched R wave progressing to fQRS) in one lead for a subject, then we only counted the change once. Figure 3 shows the location of the first change in any ECG lead for each subject.

## Discussion

Among cardiovascular subjects, we found significant ECG changes prior to PEA and asystole. Gradually decrease in heart rate, QRS prolongation, changes in QRS morphology and ST segment depression and elevation were the main findings prior to I-HCA with initial rhythms of PEA and asystole.

We found evidence that continuous ECG monitoring systems/telemetry are currently incapable of detecting important ECG changes prior to cardiac arrest and alerting clinicians of these changes. For example, the ECG system sounds an alarm when the heart rate is high or low (e.g., 130–200 or 40–50). However, it does not alert the clinician when the heart rate decreases gradually from 130 to 60 beats per min. ECG changes such as a gradual decrease in heart rate within 15 min might have an immediate prognostic impact on the timely treatments of some subjects with PEA and asystole, which may be particularly important given the fact that the survival after asystole is approximately only five percent. Our study confirms previous findings that bradycardia and/or a decreased heart rate occur in adults experiencing I-HCA [13, 14]. However, our study focused on cardiovascular patients with PEA and asystole rather than a general population that developed different types of cardiac arrhythmias including ventricular tachycardia and fibrillation.

The findings suggest that the mechanism of the decrease in heart rate prior to I-HCA might be due to an imbalance between the sympathetic and parasympathetic nervous system [15–19]. It has been reported that the chronotropic response to vagal stimulation develops gradually when sympathetic stimulation precedes vagal stimulation [20]. In the remaining subjects, the high heart rates that did not subsequently decrease could have been due to sympathetic nervous system activity that dominated parasympathetic activity.

To the best of our knowledge, this study is the first to report abnormalities in ventricular conduction including both duration and morphology of the QRS prior to I-HCA due to PEA and asystole among cardiovascular subjects. QRS prolongation has been considered a prognostic marker for mortality among patients with a variety of cardiovascular diseases [21–23]. Moreover, QRS fragmentation (fQRS) has been reported to be associated with increased mortality in patients with structural heart disease [24].

The demographic findings of our study are consistent with previous findings that age is associated with mortality after I-HCA [25] and the rate of survival to discharge was approximately 20 %. It is interesting to note that the asystole group did show greater changes in QRS morphology, ST segment depression and elevation than did the PEA group. These findings suggest that the diagnostic clues for these two conditions could be different. Larger studies would look to corroborate these findings.

An additional issue inherent with PEA is that the time of arrest may not be determined precisely because patients who experience PEA have no palpable pulse but the monitoring system may show an ECG that is compatible with life. We have identified diagnostic ECG clues that warrant patient evaluation by clinicians prior to PEA and asystole as an initial rhythm of the I-HCA. This may help shorten the response time to cardiac arrest especially with PEA. Future studies will be required to determine whether these diagnostic clues can be used as a guide for appropriate interventions.

An important clinical implication of this pilot study is increased awareness of the need to use full disclosure ECGs while monitoring two ECG leads continuously. Analysis of the full disclosure ECG is not a standard feature for all ECG monitoring systems. Typically only one or two ECG leads (e.g. leads II and V1) are displayed continuously. Because the full disclosure ECG is not displayed automatically, it is not clear whether clinicians are aware they can use this feature if it is part of their telemetry system. The presence of QRS morphology and ST segment changes prior to I-HCA in leads other than II and V1 in our study supports the importance of using available full disclosure ECGs. Identification of ECG morphology changes by analyzing full disclosure ECGs may preempt or decrease response time to I-HCA due to PEA and asystole.

In conclusion, this study has identified ECG changes using full-disclosure ECGs from continuous monitoring systems that precede I-HCA due to PEA and asystole. The identification of these indicators may be a first step in better understanding the mechanism of I-HCA due to PEA and asystole. More importantly, if these changes are more widely known by medical professionals, they may be used, in concert with better telemetry and continuous monitoring systems, to prevent adverse outcomes among hospital patients from I-HCA, including death.

Telemetry is one of the most common monitoring systems being utilized at hospitals and ECG changes from telemetry can be potentially incorporated in the Early Warning Scores (EWS). Different formats of EWS (e.g., MEWS, NEWS) predict the deteriorating condition of patients by analyzing continuously recorded data including a patient’s vital signs and neurological status. ECG changes from telemetry prior to asystole and PEA can also be used to assess patients at the risk for adverse events. Establishing the ECG predictors prior to these lethal arrhythmias through multi-center studies and creating a standard of care for using monitoring systems including access to digital data at hospitals may improve our ability to predict onset of I-HCA in high risk patients, over existing clinical risk prediction methods (e.g., EWS).

## Limitations of study

Only cardiovascular disease subjects were included in this study resulting in a small sample size. However, this more homogenous population may have served as a better indicator for the potential underlying mechanisms, as opposed to a mixed population with different underlying diseases such as trauma and cancer. Nevertheless, I-HCA is a complex public health problem, and a larger, multi-center study should be conducted to verify these results and shed more light on the mechanisms of I-HCA. The present findings should be viewed with caution before making population-based conclusions because of small sample size studied. Our study indicate the potential role for telemetry in identifying patients at the risk of I-HCA and ECG changes might add meaningful clinical data to existing predictors (e.g., vital signs).

ECG data of hospitalized patients from telemetry is not typically saved as part of the patient’s electronic health records in the majority of US hospitals [26]. Therefore, only two min of each one hour period within eight hours of I-HCA were printed. More patterns can emerge as data recording practices become more consistent and ubiquitous.
